# Automatic Identification of Patients With Unexplained Left Ventricular Hypertrophy in Electronic Health Record Data to Improve Targeted Treatment and Family Screening

**DOI:** 10.3389/fcvm.2022.768847

**Published:** 2022-04-15

**Authors:** Arjan Sammani, Mark Jansen, Nynke M. de Vries, Nicolaas de Jonge, Annette F. Baas, Anneline S. J. M. te Riele, Folkert W. Asselbergs, Marish I. F. J. Oerlemans

**Affiliations:** ^1^Department of Cardiology, University Medical Center Utrecht, Utrecht University, Utrecht, Netherlands; ^2^Department of Genetics, University Medical Center Utrecht, Utrecht University, Utrecht, Netherlands; ^3^Institute of Cardiovascular Science, Faculty of Population Health Sciences, University College London, London, United Kingdom

**Keywords:** left ventricular hypertrophy (LVH), electronic health record, anderson-fabry disease, cardiac amyloidosis, text-mining

## Abstract

**Background:**

Unexplained Left Ventricular Hypertrophy (ULVH) may be caused by genetic and non-genetic etiologies (e.g., sarcomere variants, cardiac amyloid, or Anderson-Fabry's disease). Identification of ULVH patients allows for early targeted treatment and family screening.

**Aim:**

To automatically identify patients with ULVH in electronic health record (EHR) data using two computer methods: text-mining and machine learning (ML).

**Methods:**

Adults with echocardiographic measurement of interventricular septum thickness (IVSt) were included. A text-mining algorithm was developed to identify patients with ULVH. An ML algorithm including a variety of clinical, ECG and echocardiographic data was trained and tested in an 80/20% split. Clinical diagnosis of ULVH was considered the gold standard. Misclassifications were reviewed by an experienced cardiologist. Sensitivity, specificity, positive, and negative likelihood ratios (LHR+ and LHR–) of both text-mining and ML were reported.

**Results:**

In total, 26,954 subjects (median age 61 years, 55% male) were included. ULVH was diagnosed in 204/26,954 (0.8%) patients, of which 56 had amyloidosis and two Anderson-Fabry Disease. Text-mining flagged 8,192 patients with possible ULVH, of whom 159 were true positives (sensitivity, specificity, LHR+, and LHR– of 0.78, 0.67, 2.36, and 0.33). Machine learning resulted in a sensitivity, specificity, LHR+, and LHR– of 0.32, 0.99, 32, and 0.68, respectively. Pivotal variables included IVSt, systolic blood pressure, and age.

**Conclusions:**

Automatic identification of patients with ULVH is possible with both Text-mining and ML. Text-mining may be a comprehensive scaffold but can be less specific than machine learning. Deployment of either method depends on existing infrastructures and clinical applications.

## Introduction

Left ventricular hypertrophy (LVH) is a condition characterized by thickening of the left ventricular (LV) wall and can be identified using echocardiography (defined as an LV wall thickness of >12 mm). The disease has a prevalence of ±15% in the normal population (1–3). LVH in the absence of abnormal loading conditions (i.e., hypertension or valvular disease) has an estimated prevalence of ±0.2% and is named as unexplained LVH (ULVH) or hypertrophic cardiomyopathy (HCM) (3, 4). ULVH is an important cause of sudden cardiac death and is caused by autosomal dominant genetic mutations in genes encoding proteins of the cardiac sarcomere in 40–60% of patients (5–7). Some ULVH cases are explained by a variety of rare, genetic, and non-genetic etiologies that may produce isolated or syndromic LVH, such as cardiac amyloidosis in an estimated 5–10% and Anderson-Fabry's Disease (AFD) in 0.5–1% of cases (3, 8–11). These specific etiologies are also referred to as phenocopies.

Identification of patients with ULVH is important to allow risk stratification for sudden cardiac death and screening of at-risk family members (12–14). Early identification of cardiac amyloidosis and AFD is essential to initiate targeted treatment to slow disease progression and improve patient prognosis (15–17). However, timely identification is hampered by low disease prevalence, intrinsic phenotypic heterogeneity, presence of comorbidities or absence of an indicative family history (18–22).

Electronic Health Records (EHR) consist of a variety of data including both structured tables with results from clinical investigations and unstructured text data (i.e., discharge letters, clinical consultation notes, and etcetera). Text-mining is a method to extract data from unstructured datasets while machine learning (ML) algorithms can be deployed on structured datasets. Both approaches rely on research infrastructures, however the research infrastructure for text-mining may be easier to deploy than ML because it only needs one data source (clinical discharge letters) whereas ML requires a multitude of standardized clinical measurements (i.e., laboratory values, electrocardiograms, and echocardiography). Both text-mining and ML have been proposed as methods to extract diagnoses and assist in classification of patients using real-life EHR data (23–26). In this proof-of-concept-study, we aimed to assess the performance of (i) a text-mining approach and (ii) a data-driven ML approach to identify patients with ULVH, such as amyloidosis and other phenocopies.

## Materials and Methods

### Subject Inclusion

In this single-center, retrospective study, consecutive patients referred to Department of Cardiology of the University Medical Center Utrecht (UMCU) were included. Inclusion criteria were an age ≥18 years and availability of an echocardiographic interventricular septum thickness measurement before 6 December 2019 (date of text-query deployment). This study was conducted in accordance with the principles laid out in the Declaration of Helsinki and in line with guidelines provided by ethics committees and national GDPR legislature. Due to its retrospective nature and the large number of participants, this study was exempt from the Medical Research Involving Human Subjects Act (WMO) as per judgement of the Medical Ethics Committee (18/446 and 19/222 UMCU, the Netherlands) including the requirement for informed consent. Patients who had opted out of retrospective studies were excluded.

### Study Data and Infrastructure

Using the research data platform, available data on diagnosis, demographics, electrocardiograms (ECG), and echocardiography parameters, and unstructured text were retrieved from the EHR in a standardized research data platform. The design of this infrastructure has been previously published (27). Data for the ML model were restricted to a basic set of variables on these modalities to comply with a standard diagnostic workup for patients presenting for cardiological screening and to minimize the chance of data leakage. An overview of the intended parameters, methods used to handle outliers and missingness is provided in Supplementary Table 1.

### Gold Standard (Study Outcome)

The outcome of this study was ULVH diagnosis or related phenocopies cardiac amyloidosis and AFD. Three reference lists were used to adjudicate diagnoses: first, patients with ULVH diagnosis codes were extracted from the EHR (I42.1 and I42.2, International Statistical Classification of Diseases (ICD10) codes) (28). This list was then supplemented by a retrospective list of genetically-confirmed ULVH patients from the Department of Genetics. Patients were considered genetically-confirmed if a pathogenic or likely-pathogenic variant was identified, in accordance with the 2015 American College of Medical Genetics and Genomics and the Association for Molecular Pathology Standards and guidelines for the interpretation of sequence variants (29), in one or more genes with definitive, strong or moderate evidence for an association to ULVH (by M.J. and A.F.B) (30). Third, a list of consecutive patients with cardiac amyloidosis in accordance with the recently published 2021 ESC position statement on diagnosis and treatment of cardiac amyloidosis (by M.I.F.J.O.) (18). Echocardiographic LVH was defined as a maximum wall thickness of >12 mm or a left ventricular mass indexed to body surface area >115 g/m^2^ in males and >95 g/m^2^ in females, in line with current guidelines (3, 18, 21).

### Computer Algorithms

Two computer algorithms were used in this study: one computer algorithm used text-mining, and the other used ML. The details of these algorithms are available in the Supplementary Materials. In short, the text-mining algorithm was designed using CTCue (a Boolean retrieval text-mining tool) to identify patients with ULVH, defined as LVH excluding hypertension and aortic stenosis using clinical discharge letters and notes. The ML algorithm was trained on patients with echocardiographic LVH to identify patients with ULVH. Parameters for the ML algorithm are depicted in Supplementary Table 1. As ML algorithms require training on one dataset and testing in another, the model was trained on a random selection of 80% of data (stratified by outcome) and tested in 20%. To assess the added value of text-mining, “identification by text-mining” was also investigated as a dichotomous (yes/no) variable in the ML algorithm.

### Statistical Analysis

Data are presented as counts (percentages) for count data and means ± standard deviation for normally distributed or medians (interquartile range) for non-normally distributed continuous data. Performance of the ML models was assessed on the holdout set (20% of patients, stratified on outcome) after manual review of overclassified (false-positive) and missed (false-negative) subjects. Manual review was performed by a panel of experienced cardiologists in the fields of ULVH and amyloidosis (M.I.F.J.O. and F.W.A). Qualitative assessment of reasons for misclassification by the text-mining algorithm was performed by A.S. Sensitivity, specificity, positive likelihood ratio (LHR+), and negative likelihood ratio (LHR–) were reported for the models. Positive and Negative predictive values (PPV and NPV) are provided in the supplements. All analyses were performed in R version 4.0.3 (RStudio Team, 2020) using RStudio version 1.3.1093 (31).

## Results

### Study Population

**Figure 1:**
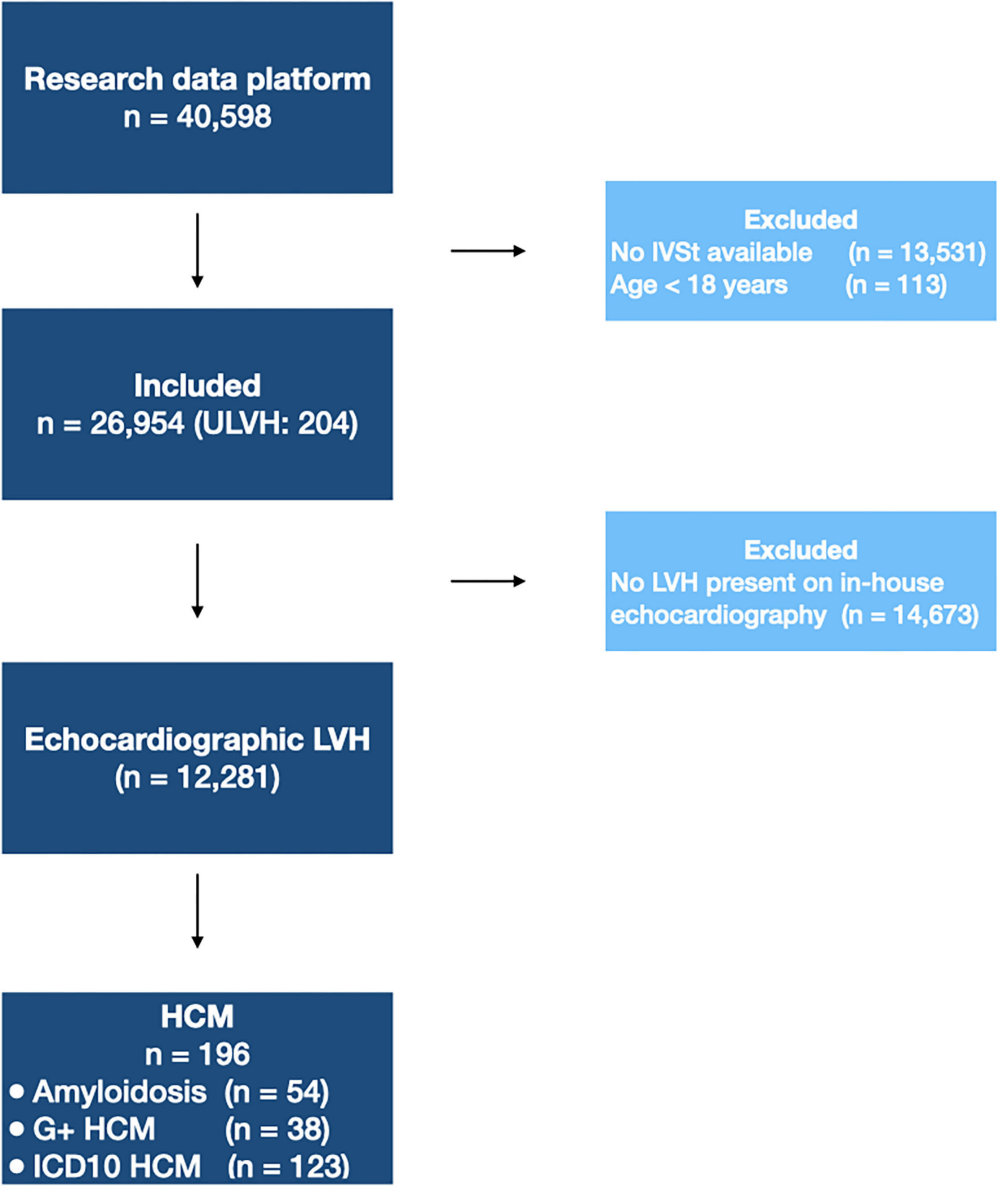
Flow diagram of patient inclusion. Flow diagram showing the patients excluded in each step. For the text-mining algorithm, 26,954 patients were included. The machine learning algorithm was trained on patients with echocardiographic LVH. IVSt, interventricular septum thickness; LVH, left ventricular hypertrophy; ULVH, Unexplained Left Ventricular Hypertrophy; HCM, Hypertrophic Cardiomyopathy; G+, genetically-confirmed; ICD10, World Health Organization International Statistical Classification of Diseases and Related Health Problems, tenth revision.

From the electronic health record (*n* = 40,598), adult patients were included in the dataset if a measurement of interventricular septal thickness (IVSt) was available (*n* = 26,954). A flow diagram of subject inclusion is provided in Figure 1. Subject characteristics are provided in Table 1. In total, 204 patients (1 in ±130) were diagnosed with ULVH, of which 56 patients were diagnosed with cardiac amyloidosis. This included 12 patients with wild-type TTR amyloidosis (median age 74.4 years, interquartile range 70.4–76.3 years) and 7 with genetic TTR amyloidosis (median age 65.8 years, interquartile range 63.9–69.3 years). Additionally, two patients were diagnosed with AFD. Genotypes of ULVH patients are summarized in Supplementary Table 2, with a total of 41 genotype positive patients and most pathogenic variants in *MYBPC3* (56%) and *MYH7* (20%). Most patients with ULVH were male (69%) and had a significantly lower mean systolic blood pressure compared to non-ULVH patients (121 vs. 129 mmHg, *p* < 0.001). ECG measurements associated with LVH were also more present in ULVH (R and S amplitudes, *p* < 0.007) as well as septal hypertrophy (1.69 vs. 1.03 cm, *p* < 0.001). All the patients with an IVSt measurement available (*n* = 26,954) were included in the text-mining dataset. To mimic clinical work-up, only patients with LVH on echocardiography were included in the ML dataset (*n* = 12,281) resulting in an exclusion of eight patients that were diagnosed with ULVH according to our gold standard (of whom two had cardiac amyloidosis, three had genetically proven ULVH, and three were identified using ICD-10 coding).

**Table 1:** Patient characteristics.

	**ULVH** **(*n* = 204)**	**No ULVH** **(*n* = 26,750)**	***p*-value**
**Demographics**			
Male sex	141 (69.1)	14,792 (55.3)	**<0.001**
Age (years)	62 [54, 70]	61 [47, 72]	0.591
Body surface area (m^2^)	1.92 [1.82, 2.10]	1.92 [1.76, 2.07]	0.053
Mean systolic blood pressure (mmHg)	121 (18)	129 (18)	**<0.001**
Mean diastolic blood pressure (mmHg)	72 (11)	74 (11)	**0.001**
**Electrocardiography**			
Atrial rate (bpm)	71 [61, 84]	72 [62, 84]	0.675
Ventricular rate (bpm)	70 [61, 82]	71 [62, 83]	0.383
P axis (°)	54 [30, 70]	54 [37, 68]	0.982
R axis (°)	19 [−38, 68]	31 [−8, 63]	0.114
T axis (°)	94 [46, 135]	51 [30, 72]	**<0.001**
PQ interval (ms)	176 [152, 206]	160 [142, 182]	**<0.001**
QRS duration (ms)	118 [98, 148]	96 [86, 110]	**<0.001**
QT interval (ms)	432 [394, 465]	396 [370, 422]	**<0.001**
QTc (Fredericia) (ms)	448 [425, 484]	417 [400, 439]	**<0.001**
R amplitude V6 (μV)	693 [364, 1,176]	937 [634, 1,274]	**<0.001**
S amplitude V2 (μV)	1,254 [649, 2,094]	1,098 [717, 1,557]	**0.007**
**Echocardiography**			
IVS thickness (mm)	16.9 [13.8, 20.0]	10.3 [8.9, 12.0]	**<0.001**
IVS/LV posterior wall ratio	1.32 [1.09, 1.69]	1.09 [0.99, 1.24]	**<0.001**
LV posterior wall thickness (mm)	13.1 [11.6, 15.4]	9.8 [8.6, 11.2]	**<0.001**
LV mass (g)	275.1 [219.6, 326.6]	177.3 [140.0, 225.6]	**<0.001**
Indexed LV mass (g/m^2^)	144.2 [116.3, 177.2]	91.8 [74.8, 114.4]	**<0.001**
LV end-diastolic diameter (mm)	45.8 (8.7)	49.3 (8.0)	**<0.001**
LV end-diastolic volume (mL)	96.9 [74.5, 119.0]	110.0 [87.6, 137.0]	**<0.001**
LV end-systolic diameter (mm)	30.0 [24.1, 36.3]	31.6 [27.2, 37.2]	**0.003**
LV end-systolic volume (mL)	39.6 [28.3, 57.7]	42.6 [30.1, 61.6]	**0.048**
LV ejection fraction (%)	55.9 [45.1, 66.5]	58.6 [49.0, 67.4]	**0.026**
LV fractional shortening (%)	32.8 [24.0, 43.5]	34.9 [27.2, 41.7]	0.226
LV outflow tract gradient (mmHg)	5.1 [3.4, 8.2]	4.0 [3.0, 5.3]	**<0.001**
Aortic valve gradient (mmHg)	8.4 [5.4, 14.3]	7.0 [5.2, 10.5]	**0.01**
LA diameter (mm)	4.5 [4.0, 5.1]	3.9 [3.5, 4.5]	**<0.001**
E/A	1.2 [0.8, 1.9]	1.0 [0.8, 1.4]	**<0.001**
Average E/e′	13.0 [9.9, 18.3]	8.1 [6.4, 10.7]	**<0.001**
Lateral E/e′	10.5 [7.0, 15.3]	6.9 [5.3, 9.3]	**<0.001**
Septal E/e′	14.7 [11.1, 19.5]	9.2 [7.2, 12.1]	**<0.001**
MV deceleration time (ms)	170 [140, 220]	180 [150, 220]	**0.009**
TAPSE (mm)	20.5 (5.4)	22.1 (5.2)	**<0.001**
**Criterium on which “outcome” was defined**			
Echocardiographic LV hypertrophy	196 (96.1)	12,085 (45.2)	**<0.001**
Maximum wall thickness >12 mm	174 (85.3)	6,010 (22.7)	**<0.001**
Indexed LV mass >115 (males) or >95 (females) g/m^2^	170 (90.4)	10,408 (45.2)	**<0.001**
Identified by CTCue population finder	159 (77.9)	8,033 (30.0)	**<0.001**

*Patient characteristics, shown as means (standard deviation), medians [interquartile range] or counts (%), stratified by ULVH diagnosis according to the reference lists (amyloidosis, genetically confirmed, and classified based on World Health Organization International Statistical Classification of Diseases and Related Health Problems, tenth revision). P-values <0.05 are shown in bold. IVS, interventricular septum; LV, left ventricular; LA, left atrial; MV; TAPSE, tricuspid annular plane systolic excursion*.

### Text-Mining

From the 26,954 subjects, the CTCue population finder algorithm flagged a total of 8,192 patients with possible ULVH, of whom 159 had ULVH and incorrectly excluding 45 ULVH cases. Patient characteristics stratified by identification by the CTCue population finder are provided in Supplementary Table 3. Patients that were identified by CTCue had characteristics that were comparable to patients with ULVH, for example with larger IVSt (1.14 vs. 1.00 cm (*p* < 0.001), larger LA dimensions [4.00 vs. 3.90 cm (*p* < 0.001) and longer PQ intervals (165 vs. 158 ms, *p* < 0.001)]. Given the identified 159 patients and missed 45 ULVH cases, Sensitivity, specificity, LHR+ and LHR– of the CTCue text-mining algorithm was 0.78, 0.67, 2.36, and 0.33, respectively. Manual reclassification revealed one additional case of ULVH which was not present in our gold standard. Reasons for under classification are provided in Supplementary Table 4, and were mostly a diagnosis of (pulmonary) hypertension (*n* = 15, 33%) and ambiguous notation of LVH (i.e., “important hypertrophy”; *n* = 7, 16%). However, in 22 patients (49%) the reason for under classification was not apparent which is discussed in the study limitations.

### Machine Learning

**Figure 2:**
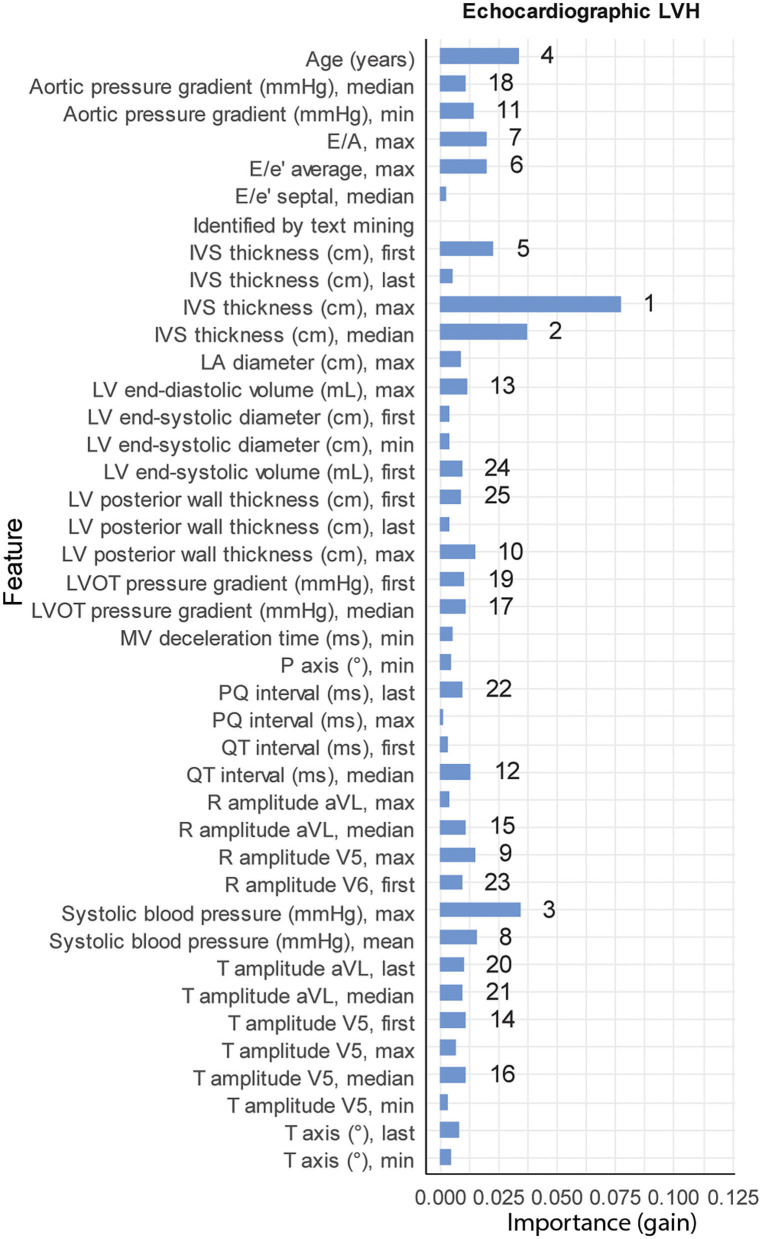
Feature importance. Relative importance for the top 25 variables of each of the three XGBoost models (41 variables in total), measured by gain. Numbers denote the rank of the top 25 variables for each model (1 being the most important). LVH, left ventricular hypertrophy.

From the 12,281 patients with echocardiographic LVH, 196 patients were previously diagnosed with ULVH. Subject characteristics stratified by echocardiographic LVH are provided in Supplementary Table 5. Patients with echocardiographic LVH were more frequently male (66.1 vs. 46.7%, *p* < 0.001), with larger LA dimensions (4.23 vs. 3.69 cm, *p* < 0.001), longer PQ interval (166 vs. 154 ms, *p* < 0.001) and longer QRS duration (102 vs. 92 ms, *p* < 0.001). The tuned hyperparameters for the trained models are provided in Supplementary Table 6. The performance of the ML models is shown in Supplementary Table 6. The test set included 39 patients with ULVH, in which ML correctly identified 10 out of 39 (26%) patients with ULVH and 2,412 (99.8% of total) without ULVH. Manual review of overclassified (false-positive, *n* = 5) cases in the test-set revealed that three were in fact true positives and missed by our golden standard list. Manual review of the misclassified (false-negatives, *n* = 29) in the test-set revealed that one case of the false-negatives was in fact sufficiently explained by hypertension resulting in a true-negative by the model. This led to a total of two false positives and 28 false negatives. Additionally, one novel case of ULVH was also identified that, in retrospect, required further work-up of LVH. Final sensitivity, specificity, LHR+ and LHR– after manual review were 0.32, 0.99, 32, and 0.69, respectively. Important variables for classification included IVSt, systolic blood pressure, and age (Figure 2).

### Added Value of Text-Mining

As shown in Supplementary Table 6, including identification by CTCue as a dichotomous variable (yes/no) did not improve performance over the baseline ML model (sensitivity, specificity, LHR+ and LHR– of 0.18, 0.99, 18, and 0.83, respectively). Coefficients and explanation of Lasso logistic regression were provided in Supplementary Table 7 and showed that including identification by CTCue as a dichotomous variable (yes/no) slightly decreased performance, correctly identifying the same number of subjects with ULVH and misclassifying one.

## Discussion

**Figure 3:**
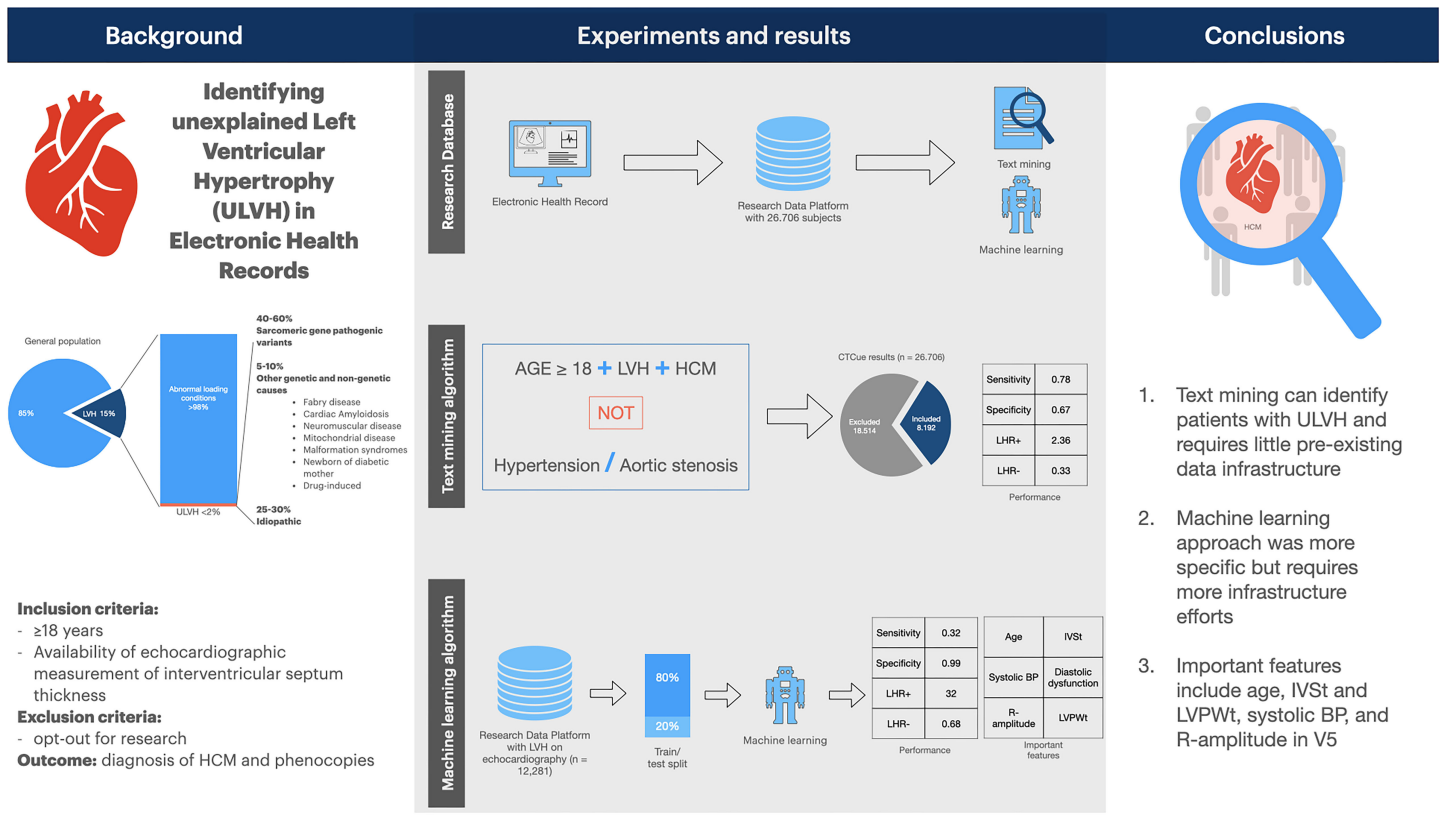
Summary figure. Summary figure of the study. BP, blood pressure. IVSt, interventricular septum thickness; LVH, left ventricular hypertrophy; LVPWt, left ventricular posterior wall thickness; LHR, likelihood ratio; ULVH, unexplained LVH.

In this study, we evaluated computer methods (text-mining and ML) in EHR data to identify patients with ULVH. These methods are feasible strategies to assist in patient screening for research databases, trial recruitment or clinical follow-up (26, 32, 33). Our results suggest that both methods can reduce the bulk of patients needed to screen with a high negative predictive value (summarized in Figure 3).

### Unexplained LVH

LVH is an echocardiographic abnormality often encountered in the normal population (±15%) (1–3). As abnormal loading conditions, such as hypertension and valvular disease are also quite common, the distinction between LVH that is sufficiently explained by these conditions and ULVH requires further investigation (3, 4). Early detection of ULVH is essential to initiate targeted treatment, for instance in AFD and cardiac amyloidosis, for risk stratification of sarcomeric ULVH and for family screening (3, 5–11). As AFD and cardiac amyloidosis are rare and therefore difficult to detect, the imperative to recognize them largely depends on availability of specific therapeutic workflows (11, 17, 20). More likely, patients present to non-experts with their initial symptoms, leading to an operational challenge to construct systems that can facilitate identification of these rare phenocopies (34). Automatic strategies to augment ULVH detection can therefore provide a systematic framework for further cardiogenetic screening of patients and relatives. With accessible EHR data approaches like text-mining or ML are practicable (35).

### Computer Algorithms

Text-mining is the process of deriving high quality information from text, in this case from clinical discharge letters. It can range from simple rule-based algorithms, to complex computer models that understand semantics and word ambiguity (26). State-of-the-art deep neural networks offer the best performance but require large amounts of language specific training data, mostly lacking for rare diseases and especially in Dutch (26, 36–38). For less-frequent diagnoses such as ULVH, rule-based methods may be a more viable option, given that the terms in text follow regular patterns (26, 32). A well-performing example is a simple classification algorithm to identify patients with systemic sclerosis using data from the EHR (32). However, the broad definition of ULVH, including phenocopies and allowing presence of concomitant abnormal loading conditions (not explaining the degree of left ventricular hypertrophy), makes precise identification of ULVH an especially challenging task (3). Furthermore, Dutch terminology for ULVH is heterogeneous, including different ways of denoting hypertrophy and spelling of hypertrophic cardiomyopathy. By using a Boolean retrieval algorithm software (CTCue), clinical criteria for ULVH were entered: excluding cases when patients had hypertension or aortic stenosis. These retrieval algorithms may be hampered by ambiguous spelling in the EHR whereas medical experts would easily identify cases when presented to them (as illustrated in the reasons for under-classification, Supplementary Table 4). In our study, text-mining identified patients with ULVH with reasonable sensitivity and LHR- which, given the epidemiology of ULVH, translates to identification of most patients with ULVH while reducing the number of patient files needed to be screened (high negative predictive value). Our results are in line with other studies using the same approach, for instance reducing the number of patients that needed to be screened for trial inclusion by 80% and a yield of 2–5% for inclusion (25). Other applications for such algorithms include retrospective cohort building, further emphasizing the supportive role of text-mining applications rather than a comprehensive solution replacing human assessment of patient inclusions (25, 39, 40). Further differentiation among ULVH types may be achieved using disease specific markers in text-mining. For instance, the search may be further targeted toward amyloidosis by following the recently published expert consensus statement, including variables such as risk factors for cardiac amyloidosis (i.e., bilateral carpal tunnel syndrome, atrioventricular block of polyneuropathy) (41). The possibility of other risk factors remains up to investigation, as a recently developed ML model identified atrial fibrillation and pericarditis to be pivotal in the selection of cardiac amyloidosis patients as well (42). Differentiation for AFD on the other hand may include variables such as kidney failure. Whether these differentiated searches for ULVH types are a viable screening method, needs to be further explored.

ML algorithms build a model based on training data to make decisions on new data without being explicitly told how to do so (learning). Our existing research data platform provided structured and standardized data to train our ML (XGBoost) algorithm (27). It identified ULVH patients with high specificity, however at the cost of sensitivity compared to the text-mining algorithm. Artificial intelligence (AI) models have previously been developed to identify patients with heart failure, or to identify patients with PLN *p.Arg14del* cardiomyopathy (43, 44). Our final model was efficient in identifying patients with ULVH, with a specificity of 0.99, LHR+ of 32 resulting in a positive predictive value of 0.72. Moreover, the model identified a previously undiagnosed patient with ULVH. A highly specific model like this would be better suited for clinical applications that require high degrees of certainty, e.g., when selecting patients to perform expensive diagnostic testing (such as Whole Genome Sequencing) or in the context of ethical considerations (whether to inform family members of a potentially inheritable phenotype) (3). As expected, coefficients were generally positive for echocardiographic characteristics of ULVH [(septal) wall thickness, LV outflow tract pressure gradient, diastolic dysfunction, and LA diameter] and negative for variables associated with abnormal loading conditions (age, blood pressure, and aortic pressure gradient).

### Infrastructure and Clinical Considerations

Big-data infrastructures improve accessibility of EHR data and methods such as machine and deep learning can model complex interactions, find new phenotype clusters, or predict prognosis (35, 45). The phenotypic data usually included in EHR systems complies with the definitions of big data and include detailed laboratory, investigations, ECG data, device data, questionnaires, and (unstructured) text (27, 35, 46). Importantly, text-mining requires little data infrastructure: it requires only one database (clinical discharge letters) and can already be implemented using a single piece of open-sourced software (47). This advantage enables easier dissemination to other centers than complex ML pipelines which often require a multitude of standardized data. Future developments for data infrastructures should focus on interoperability between EHR systems to enable validation of (complex) machine and deep learning models (35, 48).

While using text-mining and ML for patient identification and possible treatment, there are considerations limiting widespread adoption in clinical setting which including (i) algorithm performance and (ii) clinical follow-up of identified patients (45, 49). AI-algorithms may fail if selection bias occurred in dataset, reducing external validity and performance of the model. Dealing with rare diseases may for instance lead to underrepresentation in training data and subsequently be missed by AI algorithms (49). While algorithms with high positive predictive value and LHRs would accurately capture true cases, this is usually at the expense of sensitivity (33). By focussing on the needle in the haystack, the learning metric for AI algorithm must encompass a combination of both positive predictive value and sensitivity, both summarized in the F1-score. External validation in non-tertiary centers may also be necessary in rare diseases to compare effectiveness of screening algorithms. Furthermore, clinical follow-up of selected cases within a common care pathway may improve effective implementation of these algorithms compared to fragmented clinical care (50, 51).

### Study Limitations

As we used real-world data, it is possible that values in our dataset were wrong or biased due to clinical, billing, or administrative interests. Even though our center employs specialized coders to classify cardiology diagnoses (kappa of 0.78) (26), given the nature of this work, human errors in classifying disease may have added noise to the training data which is resembled by the fact that three genotype positive patients were diagnosed with ULVH without LVH. As the CTCue population finder algorithms remain proprietary (essentially a black box), this poses a major limitation in assessing algorithm shortcomings, exemplified by the fact that in 22 (49%) of patients the reason for under classification was not apparent. Use of exclusion terms for hypertension and aortic stenosis may have contributed to this, by exclusion of patients with concomitant hypertension or aortic stenosis not explanatory of the degree of hypertrophy. Conversely, the ML models were limited to structured variables. As family history is not standardized in our EHR, we could not include this in our ML models. Additionally, our manual review was restricted to misclassified subjects. The (academic) single-center study design with internal validation may limit external validity. Given GDPR compliance and the use of privacy sensitive clinical text, external validation was not available. However, our aim was not to train and publish a model that can be used, but rather to assess the feasibility of such a pipeline. Further work may be specific for data capturing systems per EHR/hospital system.

## Conclusion

In this study, we investigated two methods (text-mining and ML) to identify ULVH patients using EHR data. Our results suggest that these methods are viable options to reduce the bulk of patients needed to screen. We conclude that (i) text-mining can be easily set-up in terms of infrastructure and observed that it had reasonable sensitivity when deployed to identify patients with ULVH, (ii) ML was more specific and could be used to efficiently identify patients with ULVH though at the cost of sensitivity and infrastructure needs. Deployment depends on specific requirements of pre-existing data infrastructure, clinical framework, and ethical considerations.

## Data Availability Statement

The datasets presented in this article are not readily available because the dataset was derived from the electronic health record research data platform based on opt-out and therefore cannot be shared outside of the University Medical Center Utrecht. Requests to access the datasets should be directed to www.unravelrdp.nl.

## Ethics Statement

The studies involving human participants were reviewed and approved by Medical Ethics Committee (18/446 and 19/222 UMCU, the Netherlands). Written informed consent for participation was not required for this study in accordance with the national legislation and the institutional requirements. Written informed consent was not obtained from the individual(s) for the publication of any potentially identifiable images or data included in this article.

## Author Contributions

AS, MJ, NV, FA, and MO contributed to conception and design of the study. AS, MJ, and NV performed the experiments and wrote the first drafts of the manuscript including tables and figures. All authors contributed to manuscript revision, read, and approved submitted version.

## Funding

This work was supported by the Netherlands Cardiovascular Research Initiative with the support of the Dutch Heart Foundation (CVON2014-40 DOSIS and CVON2015-12 e-Detect to FA and AR; CVON2015-12 e-Detect young talent program to AS), Dutch Cardiovascular Alliance (2020B005 DoubleDose to FA and AB), Dutch Heart Foundation (Dekker 2015T041 to AB and MJ and Dekker 2015T058 to AR), UCL Hospitals NIHR Biomedical Research Center (to FA), UMC Utrecht Alexandre Suerman Stipend (to AS), Pfizer Netherlands (to MO), and Sanofi Genzyme (to MJ, AS, and FA).

## Conflict of Interest

MJ, AS, and FA received consultancy fees from Sanofi Genzyme. MO received consultancy fees from Alnylam, Pfizer and Novartis paid to the University Medical Center Utrecht. The remaining authors declare that the research was conducted in the absence of any commercial or financial relationships that could be construed as a potential conflict of interest.

## Publisher's Note

All claims expressed in this article are solely those of the authors and do not necessarily represent those of their affiliated organizations, or those of the publisher, the editors and the reviewers. Any product that may be evaluated in this article, or claim that may be made by its manufacturer, is not guaranteed or endorsed by the publisher.
