# Supplementary material for: Automatic Identification of Patients With Unexplained Left Ventricular Hypertrophy in Electronic Health Record Data to Improve Targeted Treatment and Family Screening
Source: Front Cardiovasc Med. 2022 Apr 15;9:768847. doi: 10.3389/fcvm.2022.768847 (PMC9051030; doi:10.3389/fcvm.2022.768847)
Supplement: Supplementary file 1 [file Data_Sheet_1.pdf]

Automatic identification of patients with unexplained left ventricular hypertrophy in electronic health record data to improve targeted treatment and family screening

Sammani, Jansen et al. (2021)

Supplements

Supplemental Methods

Text-mining

For the text mining method, an algorithm was created using the software program: CTcue population finder version 2.0.12 (CTcue, Amsterdam, The Netherlands). This tool uses Boolean retrieval method to search through unstructured EHR data such as clinical discharge letters and in-hospital consultations. The output of the tool is a list of flagged patients that meet the inclusion criteria or the query using a proprietary (black box) algorithm. The query was designed to identify patients with unexplained LVH, defined as LVH excluding hypertension and aortic stenosis and can be summarised as: ([Age > 17] AND [LVH-synonyms OR ULVH-synonyms] AND [patient at cardiology]) NOT ([hypertension-synonyms] OR [aortic stenosis-synonym]). Synonyms included suggestions by the built-in synonym expander supplemented with commonly used synonyms and abbreviations.

Machine learning algorithm

Within subjects with LVH on echocardiography, an XGBoost algorithm was trained. The model was trained on a random selection of 80% of data (train set, stratified on outcome). Echocardiographic LVH was defined as a maximum wall thickness of >12 mm or a left ventricular mass indexed to body surface area >115 in males and >95 in females, in line with current guidelines.[3,21,31] An additional model was built using identification by CTCue as a dichotomous variable (yes/no) to address added value of CTCue in identifying ULVH within this subset dataset with LVH. XGBoost is an ensemble ML algorithm that uses extreme gradient boosting framework to convert a set of weak tree classifiers into a single strong classifier. It iterates through a process of re-weighting, adding terminal node penalisation (gamma) to allow variability in the numbers of terminal nodes per tree, additional regularisation of terminal node weights, Newton boosting to fit subsequent trees and column subsampling as an additional randomisation parameter.[1,2] Hyperparameters were tuned using consecutive 5-fold cross-validated grid-searches (provided in the Supplemental Methods) with the *caret* package.[3] The model was tested in 20% of the data. To provide a readily interpretable model, logistic regression was fitted on the train set using the top 50 best performing variables. Missing data were imputed using iterative Random forest imputations consisting of 100 trees per forest and a maximum of 10 iterations, using the *missForest* package.[4] Logistic regression was performed using 5-fold cross-validated Lasso regression to attenuate multicollinearity effects, using the *caret* and *glmnet* packages.[5]

1. Friedman JH. Greedy function approximation: A gradient boosting machine. Ann Stat [Internet]. 2001;29:1189–232. Available from: http://projecteuclid.org/euclid.aos/1013203451

2. Chen T, Guestrin C. XGBoost: A scalable tree boosting system. Proc ACM SIGKDD Int Conf Knowl Discov Data Min [Internet]. New York, NY, USA: ACM; 2016. p. 785–94. Available from: https://dl.acm.org/doi/10.1145/2939672.2939785

3. Kuhn M. Building predictive models in R using the caret package. J Stat Softw [Internet]. 2008;28:1–26. Available from: http://www.jstatsoft.org/v28/i05/

4. Stekhoven DJ, Buhlmann P. MissForest--non-parametric missing value imputation for mixed-type data. Bioinformatics [Internet]. 2012;28:112–8. Available from: https://academic.oup.com/bioinformatics/article-lookup/doi/10.1093/bioinformatics/btr597

5. Friedman J, Hastie T, Tibshirani R. Regularization paths for generalized linear models via coordinate descent. J Stat Softw [Internet]. 2010;33:1–22. Available from: http://www.ncbi.nlm.nih.gov/pubmed/20808728

XGBoost hyperparameter tuning

Hyperparameter tuning was performed using grid searches in five steps, as detailed on Pelkoja (2018). "Visual XGBoost Tuning with caret." (Retrieved 19-10-2020, from https://www.kaggle.com/pelkoja/visual-xgboost-tuning-with-caret).

Step 1. Temporarily fixing learning rate

Number of iterations	200 to 1000, per 50
Learning rate	0.025, 0.05, 0.1, 0.3
Maximum tree depth	2, 3, 4, 5, 6
Gamma	0
Subsample ratio of columns	1
Subsample ratio of rows	1
Minimum sum of instance weight required in a child	1

Step 2. Maximum tree depth & minimum sum of instance weights required in a child

Number of iterations	50 to 1000, per 50
Learning rate	Step 1 best
Maximum tree depth	2-4 step 1 best was 2,<br>otherwise step 1 best -1 to step 1 best +1
Gamma	0
Subsample ratio of columns	1
Subsample ratio of rows	1
Minimum sum of instance weight required in a child	1, 2, 3

Step 3. Subsample ratios

Number of iterations	50 to 1000, per 50
Learning rate	Step 1 best
Maximum tree depth	Step 2 best
Gamma	0
Subsample ratio of columns	0.4, 0.6, 0.8, 1.0
Subsample ratio of rows	0.5, 0.75, 1.0
Minimum sum of instance weight required in a child	Step 2 best

Step 4. Gamma

Number of iterations	50 to 1000, per 50
Learning rate	Step 1 best
Maximum tree depth	Step 2 best
Gamma	0, 0.05, 0.1, 0.5, 0.7, 0.9, 1.0
Subsample ratio of columns	Step 3 best
Subsample ratio of rows	Step 3 best
Minimum sum of instance weight required in a child	Step 2 best

Step 5. Reducing the learning rate eta & determining number of iterations

Number of iterations	100 to 10,000, per 100
Learning rate	0.01, 0.015, 0.025, 0.05, 0.1
Maximum tree depth	Step 2 best
Gamma	Step 4 best
Subsample ratio of columns	Step 3 best
Subsample ratio of rows	Step 3 best
Minimum sum of instance weight required in a child	Step 2 best

Final model

Number of iterations	Step 5 best
Learning rate	Step 5 best
Maximum tree depth	Step 2 best
Gamma	Step 4 best
Subsample ratio of columns	Step 3 best
Subsample ratio of rows	Step 3 best
Minimum sum of instance weight required in a child	Step 2 best

Supplemental Tables

Supplemental Table 1. Parameters & outlier handling

Variable	Outlier handling	Values	Missingness
**Demographics**			
Sex	-	-	0.000
Age (years)	Values < 18 excluded	At last echo	0.000
Mean systolic blood pressure (mmHg)	Values < 1 & > 300 excluded	Min, mean, max	0.255
Mean diastolic blood pressure (mmHg)	Values < 1 excluded	Min, mean, max	0.255
Body surface area (m^2)	Values > 5 & < 0.5 excluded	First, last, min, median, max	0.102
**Electrocardiography**			
Atrial rate (bpm)	-	First, last, min, median, max	0.013
Ventricular rate (bpm)	-	First, last, min, median, max	0.013
P axis (°)	-	First, last, min, median, max	0.037
R axis (°)	-	First, last, min, median, max	0.014
T axis (°)	-	First, last, min, median, max	0.014
PQ interval (ms)	-	First, last, min, median, max	0.037
QRS duration (ms)	-	First, last, min, median, max	0.013
QT interval (ms)	-	First, last, min, median, max	0.013
QTc (Bazett) (ms)	-	First, last, min, median, max	0.013
QTc (Fredericia) (ms)	-	First, last, min, median, max	0.047
P peak amplitude (II)	-	first, last, min, median, max	0.013
PP peak amplitude (V1)	-	first, last, min, median, max	0.013
Q peak amplitude (aVL, V5, V6)	-	First, last, min, median, max	0.013
Q peak area (I-III, aVF/-L, V5, V6)	-	First, last, min, median, max	0.013
R max. amplitude (I, aVL, V5, V6)	-	First, last, min, median, max	0.013
S max. amplitude (III, aVR, V1-3)	-	First, last, min, median, max	0.013
ST minimum (I, aVL, V5, V6)	-	First, last, min, median, max	0.013
T peak amplitude (I, aVL, V5, V6)	-	First, last, min, median, max	0.013
**Echocardiography**			
IVS thickness (cm)	Values < 0.2 & >4.0 excluded	First, last, min, median, max	0.010
IVS/LV posterior wall ratio	Values < 0.2 & >4.0 excluded	First, last, min, median, max	0.031
LV posterior wall thickness (cm)	Values < 0.1 & >5.0 excluded	First, last, min, median, max	0.016
LV mass (g)	Values < 20 & > 400 excluded	First, last, min, median, max	0.023
Indexed LV mass (g/m^2)	Values < 10 & > 300 excluded	First, last, min, median, max	0.132
LV end-diastolic diameter (cm)	Values < 0 & > 15 excluded	First, last, min, median, max	0.012
LV end-diastolic volume (mL) *	Values <30 & > 1000 excluded	First, last, min, median, max	0.012
LV end-systolic diameter (cm)	Values < 0 & > 10 excluded	First, last, min, median, max	0.200
LV end-systolic volume (mL) *	Values < 5 & > 500 excluded	First, last, min, median, max	0.151
LV ejection fraction (%) *	Values < 10 & > 80 excluded	First, last, min, median, max	0.193
LV fractional shortening (%)	Values < 5 & > 80 excluded	First, last, min, median, max	0.206
LV outflow tract gradient (mmHg)	Values < 0 & > 200 excluded	First, last, min, median, max	0.100
Aortic valve gradient (mmHg)	Values < 0 & > 200 excluded	First, last, min, median, max	0.073
LA dimension (cm)	Values < 1 & > 9.9 excluded	First, last, min, median, max	0.409
LA volume (mL)	Excluded (missingness)		0.617
Indexed LA volume (mL/m^2)	Excluded (missingness)		0.629
E/A	Values < 0 & > 5 excluded	First, last, min, median, max	0.143
Average E/e'	Values < 0 & > 40 excluded	First, last, min, median, max	0.260
Lateral E/e'	Values < 0 & > 40 excluded	First, last, min, median, max	0.252
Septal E/e'	Values < 0 & > 40 excluded	First, last, min, median, max	0.253
MV deceleration time (s)	Values < 0.030 & 0.600 excluded	First, last, min, median, max	0.257
TAPSE (cm)	Values < 1 & > 40 excluded	First, last, min, median, max	0.232

List of the variables (a priori) intended for modelling, showing outlier handling strategies, values taken from longitudinal measurements and missingness. Missingness >0.50 is indicated in red.

* Taken from available methods, in the following order: (i) Modified Simpson, (ii) 3D-methods, (iii) other biplane methods, (iv) Teichholz's/cubed formula.

IVS, interventricular septum; LV, left ventricular; LA, left atrial; MV, mitral valve; TAPSE, tricuspid annular plane systolic excursion.

Supplemental Table 2. Genotypes

	All G+ HCM (n = 41)		G+ Echocardiographic LVH (n = 38)		G+ Text mining (n = 35)	
	**P**	**LP**	**P**	**LP**	**P**	**LP**
**Definitive**						
MYBPC3	22 (56.4) *†		21 (56.8) *†		19 (57.6) *†	
MYH7	4 (11.1)	3 (8.6)	3 (9.1)	3 (9.1)	4 (13.3)	3 (10.3)
TNNT2		1 (2.8)		1 (2.9)		0 (0.0)
TNNI3	1 (2.8)		1 (2.9)		0 (0.0)	
MYL3	1 (2.9)	1 (2.9)	1 (3.0)	1 (3.0)	1 (3.4)	1 (3.4)
MYL2	1 (2.8)		0 (0.0)		1 (3.3)	
GLA (Fabry disease)	2 (5.6)		2 (5.9)		2 (6.7)	
TTR (amyloidosis)	1 (2.7)		1 (2.9)		1 (3.2)	
**Moderate**						
CSRP3	4 (11.1) *		4 (11.8) *		4 (13.3) *	
ACTN2		2 (5.6) †		2 (5.9) †		1 (3.3) †

Number of patients with pathogenic or likely pathogenic variants (per gene), showing variants identified in the overall study population and in the subpopulations identified by selecting patients with echocardiographic left ventricular hypertrophy or using text mining.

* including one patient with a pathogenic variant in *MYBPC3* and a pathogenic variant in *CSRP3*; † including one patient with a pathogenic variant in *MYBPC3* and a likely pathogenic variant in *ACTN2*.

G+, genetically-confirmed; HCM, hypertrophic cardiomyopathy; P, pathogenic; LP, likely pathogenic; LVH, left ventricular hypertrophy

Supplemental Table 3. Baseline characteristics stratified by CTCue

	Identified by CTCue (n = 8,123)	Not identified by CTCue (n = 18,583)	p-value
**Demographics**			
Sex (male)	4744 (58.4)	10027 (54.0)	**<0.001**
Age (years)	63.43 [50.81, 73.01]	59.86 [45.20, 71.52]	**<0.001**
Body surface area (m^2)	1.93 [1.78, 2.09]	1.91 [1.75, 2.06]	**<0.001**
Systolic blood pressure (mmHg)	132.15 (17.83)	127.42 (17.70)	**<0.001**
Diastolic blood pressure (mmHg)	75.71 (10.77)	73.74 (10.40)	**<0.001**
**Electrocardiography**			
Atrial rate (bpm)	70.00 [62.00, 81.00]	72.00 [63.00, 85.00]	**<0.001**
Ventricular rate (bpm)	70.00 [62.00, 80.00]	72.00 [63.00, 84.00]	**<0.001**
P axis (°)	54.00 [36.00, 67.00]	54.00 [38.00, 68.00]	**0.01**
R axis (°)	24.00 [-13.00, 58.00]	34.00 [-6.00, 65.00]	**<0.001**
T axis (°)	53.00 [31.00, 75.00]	50.00 [29.00, 71.00]	**<0.001**
PQ interval (ms)	164.00 [146.00, 186.00]	158.00 [140.00, 180.00]	**<0.001**
QRS duration (ms)	98.00 [88.00, 110.00]	96.00 [86.00, 110.00]	**<0.001**
QT interval (ms)	398.00 [376.00, 424.00]	394.00 [368.00, 422.00]	**<0.001**
QTc (Fredericia) (ms)	417.00 [401.75, 439.00]	416.00 [399.00, 440.00]	**0.034**
**Echocardiography**			
IVS thickness (cm)	1.14 [0.98, 1.32]	1.00 [0.86, 1.14]	**<0.001**
IVS/LV posterior wall ratio	1.14 [1.02, 1.31]	1.07 [0.97, 1.21]	**<0.001**
LV posterior wall thickness (cm)	1.06 [0.92, 1.20]	0.96 [0.84, 1.08]	**<0.001**
LV mass (g)	195.66 [154.39, 246.17]	171.43 [135.78, 218.29]	**<0.001**
Indexed LV mass (g/m^2)	99.82 [81.06, 123.98]	88.10 [72.33, 109.34]	**<0.001**
LV end-diastolic diameter (cm)	4.83 (0.76)	4.97 (0.83)	**<0.001**
LV end-diastolic volume (mL)	106.69 [84.82, 133.81]	111.27 [88.53, 138.11]	**<0.001**
LV end-systolic diameter (cm)	3.12 [2.68, 3.65]	3.17 [2.74, 3.74]	**<0.001**
LV end-systolic volume (mL)	42.49 [29.67, 60.52]	42.39 [30.16, 62.04]	**0.006**
LV ejection fraction (%)	58.81 [50.17, 68.37]	59.86 [49.36, 69.62]	0.137
LV fractional shortening (%)	34.90 [27.25, 41.92]	34.81 [26.67, 41.58]	**0.029**
LV outflow tract gradient (mmHg)	3.60 [2.64, 4.89]	3.55 [2.59, 4.68]	**<0.001**
Aortic valve gradient (mmHg)	6.97 [5.05, 10.90]	6.37 [4.78, 9.03]	**<0.001**
LA dimension (cm)	4.00 [3.56, 4.54]	3.90 [3.45, 4.45]	**<0.001**
E/A	0.95 [0.73, 1.32]	1.07 [0.79, 1.46]	**<0.001**
Average E/e'	8.45 [6.75, 11.21]	7.89 [6.28, 10.51]	**<0.001**
Lateral E/e'	7.22 [5.58, 9.74]	6.72 [5.23, 9.17]	**<0.001**
Septal E/e'	9.62 [7.57, 12.80]	8.96 [7.04, 11.91]	**<0.001**
MV deceleration time (s)	0.19 [0.16, 0.23]	0.18 [0.15, 0.21]	**<0.001**
TAPSE (cm)	2.21 (0.53)	2.19 (0.54)	**0.022**
**Outcome criteria**			
Left ventricular hypertrophy	4767 (58.7)	7090 (38.2)	**<0.001**
Maximum wall thickness ≥13 mm	2491 (30.7)	2483 (13.4)	**<0.001**
LV mass/BSA >115 (male), >95 (female) g/m^2	4241 (57.0)	6336 (40.2)	**<0.001**
ULVH diagnosis	159 (2.0)	45 (0.2)	**<0.001**
Amyloidosis	37	19	
G+ HCM	35	6	
ICD10	100	26	

Subject characteristics, shown as means (standard deviation), medians [interquartile range] or counts (%), stratified by identification by text mining. P-values <0.05 are shown in bold. IVS, interventricular septum; LV, left ventricular;

LA, left atrial; MV; TAPSE, tricuspid annular plane systolic excursion; ICD10, World Health Organization International Statistical Classification of Diseases and Related Health Problems, tenth revision; ULVH:Unexplained Left Ventricular Hypertrophy.

Supplemental Table 4. Qualitative assessment of under classification by CTCue

LVH not mentioned	Ambigious writing	Hypertension	Aortic stenosis	LVH clearly mentioned	Notes	Missed, and can be explained logically	Missed, without apparent explanation	HCM diagnosed by cardiologist?
	yes				"duidelijke hypertrofie", but left ventricle not mentioned	yes	no	yes
				yes	hypertrofie linkerventrikel, should not have been missed	no	yes	yes
				yes	"linkerventrikelhypertrofie " should not have been missed	no	yes	yes
				yes		no	yes	yes
yes						yes	no	no
		yes		yes	Restrictive CMP with amyloid and LVH, also essentiele hypertensie	yes	no	no
				yes	hypertensie: nee	no	yes	yes
yes					Cardio file without cardio consultation, no mention of LVH	yes	no	no
				yes		no	yes	yes
	yes	yes			First controls: geen HOCM, but developed it later on	yes	no	yes
		yes	yes	yes	No HCM but explained LVH	yes	no	no
				yes	geringe LVH, hypertensie: nee	no	yes	yes
				yes		no	yes	yes
				yes	linker ventrikelhypertropfie	no	yes	yes
	yes				belangrijke hypertrofie	yes	no	yes
	yes				concentrische hypertrofie van de linkerventrikelwand	yes	no	yes
		yes		yes	HCM	yes	no	yes
				yes	HOCM	no	yes	yes
				yes	HCM	no	yes	yes
				yes	hypertrophische cardiomyopathiemutatie and LVH mentioned in letters	no	yes	yes
				yes	HCM	no	yes	yes
				yes	hypertrofische cardiomyopathie	no	yes	yes
				yes	hypertrofische cardiomyopathie	no	yes	yes
	yes				AL amyloidose met RCM "en wanden vrij fors zijn"	yes	no	no
				yes	hypertrofische cardiomyopathie	no	yes	yes
				yes	HCM	no	yes	yes
		yes	yes	yes	"lichte hypertrofie"	yes	no	no
		yes		yes	LVH	yes	no	yes
		yes		yes	hypertrofe cardiomyopathie; pulmonale hypertensie	yes	yes	yes
				yes	hypertrofische obstructieve cardiomyopathie	no	yes	yes
	yes				biventriculaire hypertrofie	yes	no	yes
		yes		yes	linker ventrikel hypertrofie	yes	no	yes
		yes		yes	HCM and hypertension	yes	no	yes
no					restrictieve cmp met geringe deel hypertrofie	yes	no	no
				yes	beginnende LVH	no	yes	yes
		yes		yes	hypertensie not negated in text	yes	no	yes
				yes	hypertrofe obstructieve cardiomyopathie	no	yes	yes
				yes	hypertrofische cardiomyopathie	no	yes	yes
				yes	hcm	no	yes	yes
	yes				hypertrofische biventriculaire cardiomyopathie	yes	no	yes
		yes		yes	pulmonale hypertensie; toont duidelijke LVH	yes	no	yes
		yes		yes	pulmonale hypertensie	yes	no	yes
		yes		yes	hypertensieve CMP	yes	no	no
		yes		yes	hypertrofische cardiomyopathie	yes	no	yes
		yes		yes	hypertrofische cardiomyopathie	yes	no	yes

Supplemental Table 5. Baseline characteristics stratified by left ventricular hypertrophy

	Left ventricular hypertrophy (n = 11857)	No left ventricular hypertrophy (n = 14849)	p-value
**Demographics**			
Sex (male)	7841 (66.1)	6930 (46.7)	**<0.001**
Age (years)	66.30 [54.35, 75.35]	56.29 [41.03, 68.34]	**<0.001**
Body surface area (m^2)	1.94 [1.79, 2.09]	1.88 [1.74, 2.04]	**<0.001**
Systolic blood pressure (mmHg)	129.72 (18.46)	128.42 (17.32)	**<0.001**
Diastolic blood pressure (mmHg)	74.18 (10.79)	74.63 (10.36)	**0.003**
**Electrocardiography**			
Atrial rate (bpm)	72.00 [62.00, 85.00]	72.00 [63.00, 84.00]	0.134
Ventricular rate (bpm)	71.00 [62.00, 83.00]	71.00 [63.00, 83.00]	0.633
P axis (°)	54.00 [35.00, 68.00]	55.00 [38.00, 67.00]	0.098
R axis (°)	17.00 [-22.00, 57.00]	40.00 [4.00, 66.00]	**<0.001**
T axis (°)	56.00 [29.00, 89.00]	48.00 [30.00, 65.00]	**<0.001**
PQ interval (ms)	166.00 [146.00, 190.00]	154.00 [138.00, 174.00]	**<0.001**
QRS duration (ms)	102.00 [92.00, 128.00]	92.00 [84.00, 102.00]	**<0.001**
QT interval (ms)	404.00 [378.00, 436.00]	390.00 [366.00, 414.00]	**<0.001**
QTc (Fredericia) (ms)	425.00 [406.00, 454.00]	411.00 [396.00, 429.00]	**<0.001**
**Echocardiography**			
IVS thickness (cm)	1.20 [1.05, 1.38]	0.93 [0.82, 1.04]	**<0.001**
IVS/LV posterior wall ratio	1.15 [1.02, 1.32]	1.05 [0.96, 1.18]	**<0.001**
LV posterior wall thickness (cm)	1.11 [0.99, 1.25]	0.90 [0.80, 1.00]	**<0.001**
LV mass (g)	229.74 [198.44, 274.01]	145.78 [121.43, 171.08]	**<0.001**
Indexed LV mass (g/m^2)	115.55 [102.54, 136.77]	75.63 [65.10, 84.90]	**<0.001**
LV end-diastolic diameter (cm)	5.18 (0.94)	4.72 (0.63)	**<0.001**
LV end-diastolic volume (mL)	123.63 [95.20, 156.26]	102.16 [83.59, 123.08]	**<0.001**
LV end-systolic diameter (cm)	3.43 [2.89, 4.18]	3.00 [2.62, 3.40]	**<0.001**
LV end-systolic volume (mL)	51.17 [34.76, 79.80]	37.47 [27.34, 50.23]	**<0.001**
LV ejection fraction (%)	55.78 [42.91, 66.99]	62.02 [54.12, 70.62]	**<0.001**
LV fractional shortening (%)	32.54 [22.62, 40.76]	36.17 [29.91, 42.32]	**<0.001**
LV outflow tract gradient (mmHg)	4.09 [2.98, 5.65]	3.97 [3.02, 5.16]	**<0.001**
Aortic valve gradient (mmHg)	8.06 [5.68, 14.27]	6.55 [5.02, 8.85]	**<0.001**
LA dimension (cm)	4.23 [3.78, 4.82]	3.69 [3.30, 4.11]	**<0.001**
E/A	0.96 [0.71, 1.37]	1.08 [0.81, 1.44]	**<0.001**
Average E/e'	9.23 [7.09, 12.69]	7.44 [6.03, 9.32]	**<0.001**
Lateral E/e'	7.79 [5.85, 10.92]	6.35 [5.05, 8.20]	**<0.001**
Septal E/e'	10.44 [7.97, 14.57]	8.39 [6.76, 10.63]	**<0.001**
MV deceleration time (s)	0.19 [0.15, 0.23]	0.18 [0.15, 0.21]	**<0.001**
TAPSE (cm)	2.12 (0.57)	2.27 (0.51)	**<0.001**
**Outcome criteria**			
Identified by CTCue population finder	4767 (40.2)	3356 (22.6)	**<0.001**
ULVH diagnosis	193 (1.6)	11 (0.1)	**<0.001**
Amyloidosis	53	3	
G+ HCM	38	3	
ICD10	121	5	

Subject characteristics, shown as means (standard deviation), medians [interquartile range] or counts (%), stratified by presence of echocardiographic left ventricular hypertrophy (maximum wall thickness of >12 mm or a left ventricular mass indexed to body surface area >115 in males and >95 in females). P-values <0.05 are shown in bold.

IVS, interventricular septum; LV, left ventricular; LA, left atrial; MV; TAPSE, tricuspid annular plane systolic excursion; ICD10, World Health Organization International Statistical Classification of Diseases and Related Health Problems, tenth revision.

Supplemental Table 6. Performance measures XGBoost

	Echocardiographic LVH (n = 2,456)	Echocardiographic LVH (text mining as variable) (n = 2,456)	Text mining (n = 1,637)
**Before manual review**			
Sensitivity	0.2564	0.1795	0.1290
Specificity	0.9979	0.9979	0.9988
Positive predictive value	0.6667	0.5833	0.6667
Negative predictive value	0.9881	0.9869	0.9834
Likelihood ratio +	26	18	13
Likelihood ratio -	0.75	0.83	0.88
**After manual review**			
Sensitivity	0.32		
Specificity	0.99		
Positive predictive value	0.72		
Negative predictive value	0.99		
Likelihood ratio +	32		
Likelihood ratio -	0.69		

Performance of the three hypertuned XGBoost models on the holdout set (20% of total subjects identified through each method), before and after reclassification by manual review. LVH, left ventricular hypertrophy.

Supplemental Table 7. Performance measures Lasso regression

	Coefficients without text mining variable	Coefficients with text mining variable
Intercept	-10.2	-12.5
Age (years)	-0.0212	-0.0186
Systolic blood pressure (mmHg), max	-0.0157	-0.0165
Systolic blood pressure (mmHg), mean	-0.0117	-0.0151
P axis (°), last	-1.64E-04	-
T axis (°), first	2.17E-03	2.30E-03
T axis (°), last	3.03E-03	3.67E-03
PQ interval (ms), median	2.76E-03	2.24E-03
QT interval (ms), median	0.0118	0.0103
QTC Fredericia (ms), last	1.50E-03	3.17E-03
P area II, max	-	2.54E-06
P area II, median	4.04E-04	4.24E-04
Q amplitude aVL, max	5.73E-05	1.02E-04
R amplitude aVL, median	-2.25E-04	-3.11E-04
R amplitude I, first	2.07E-04	2.02E-04
R amplitude V6, first	-4.10E-04	-4.18E-04
S amplitude V3, first	-5.76E-05	-9.40E-05
T amplitude aVL, last	-2.35E-04	-2.30E-04
T amplitude aVL, median	-4.89E-04	-3.03E-04
T amplitude V5, first	-5.05E-04	-5.29E-04
T amplitude V5, median	-6.49E-04	-4.54E-04
IVS thickness (cm), first	0.274	0.472
IVS thickness (cm), max	1.02	0.768
IVS thickness (cm), median	0.816	0.699
LV posterior wall thickness (cm), first	0.556	0.259
LV posterior wall thickness (cm), max	1.04	0.705
LV posterior wall thickness (cm), median	0.152	0.903
LV end-diastolic diameter, median	-8.05E-03	-
LV end-diastolic volume (mL), max	-	-2.51E-03
LV end-diastolic volume (mL), median	-5.21E-03	-2.25E-03
LV end-systolic volume (mL), first	-7.33E-03	-5.66E-03
LVOT pressure gradient (mmHg), first	7.41E-03	0.0103
LVOT pressure gradient (mmHg), median	1.86E-03	2.60E-03
Aortic pressure gradient (mmHg), median	-0.0351	-0.0381
LA diameter (cm), max	0.187	0.225
E/A, max	0.292	0.305
E/e' average, max	7.24E-03	0.0129
E/e' lateral, median	9.83E-03	0.0121
E/e' septal, max	0.0309	0.0297

MV deceleration time (ms), min	-0.386	-0.814
Identified by text mining	-	1.60

Coefficients of the logistic Lasso regression fitted to the train data of the subjects with echocardiographic left ventricular hypertrophy (n = 9,825). Coefficients correspond to each unit increase of variable. The Lasso logistic regression fitted on the subjects with echocardiographic LVH (best lambda = 0.001) using the top 50 XGBoost variables correctly identified 6 out of 39 subjects with and 2,412 out of 2,417 subjects without ULVH (sensitivity 0.154, specificity 0.998, PPV 0.545, NPV 0.987). Inclusion of text mining as a variable (best lambda = 0.001) slightly decreased performance, correctly identifying the same numbers of subjects with ULVH but misclassifying one additional subject without ULVH (2411 out of 2417; specificity 0.998, PPV 0.500).
